# Investigation of osmotic shock effect on pulsed electric field treated *S. cerevisiae* yeast cells

**DOI:** 10.1038/s41598-023-37719-4

**Published:** 2023-06-29

**Authors:** Greta Gančytė, Povilas Šimonis, Arūnas Stirkė

**Affiliations:** 1 Laboratory of Bioelectrics, Center for Physical Sciences and Technology, State Research Institute, Sauletekio Ave. 3, 10257 Vilnius, Lithuania; 2 Micro and Nanodevices Laboratory, Institute of Solid State Physics, University of Latvia, Kengaraga Str. 8, Riga, 1063 Latvia

**Keywords:** Biochemistry, Biological techniques, Biophysics, Biotechnology, Chemical biology

## Abstract

Pulsed electric field (PEF) treatment is known to cause plasma membrane permeabilization of microorganisms, an effect known as electroporation. PEF treatment is very attractive since it can achieve permeabilization with or without lethal damage in accordance with desired results. This study aimed to expand the accomplishment of electroporation outcomes by applying sudden post-PEF osmotic composition change of the media. Changes in yeast cells’ viability, size and plasma membrane regeneration rate were evaluated. However, we still have questions about the intracellular biochemical processes responsible for plasma membrane recovery after electroporation. Our suggested candidate is the high osmolarity glycerol (HOG) kinase pathway. The HOG pathway in *Saccharomyces cerevisiae* yeasts is responsible for volume recovery after dangerous shape modifications and intracellular water disbalance caused by environmental osmotic pressure changes. Thus, we evaluated the HOG pathway inactivation effect on *S. cerevisiae’s* reaction to PEF treatment. Results showed that Hog1 deficient *S. cerevisiae* cells were considerably more sensitive to electric field treatment, confirming a link between the HOG pathway and *S. cerevisiae* recovery process after electroporation. By suddenly changing the osmolarity of the media after PEF we influenced the cells’ plasma membrane recovery rate, severity of permeabilization and survivability of yeast cells. Studies of electroporation in combination with various treatments might improve electric field application range, efficiency, and optimization of the process.

## Introduction

Throughout the years, manipulation of cell integrity has been proven to be a valuable tool in medicine^1–3^, biotechnology and the food industry field^4–6^. Electroporation is a novel and rapidly developing technology used to increase permeability by the creation of pores in cells` membranes by pulsed electric field (PEF) exposure^7^. Increased cell membrane permeability is the critical requirement for desired molecule exchange. Electroporation severity depends on parameters such as intensity of the electric field strength, pulse duration, and the number of pulses applied^7–9^.

Manipulation of electric field parameters can result in reversible or irreversible electroporation effects. The process of reversible electroporation involves the regeneration of pores over time, and the cells remain viable even after the electric pulse^7^. The most popular method and a good example of reversible electroporation application would be the transformation of various microorganisms^10–12^. Moreover, the introduction of target substances, such as cryoprotectants to improve freezing tolerance is a nice illustration of reversible electroporation as well^13^. Whereas, irreversible electroporation occurs when most pores either fail to reseal or reseal too slowly to maintain viability, thus, cells disintegrate^7^. There are a few fields of irreversible electroporation application: tissue ablation^14,15^, extraction of target substances^4^, and inactivation of microorganisms^16^.

Yeast cells possess a plasma membrane and a rigid outer cell wall that provide structural integrity and protection against external stressors. The cell wall, due to its rigidity, is a major component of yeast's response to osmotic pressure^17^. Therefore, understanding both the reversible and irreversible effects of PEF on both the plasma membrane and the cell wall of yeasts is essential for a fundamental understanding of PEF treatment. We utilized osmotic changes in media after PEF to illuminate the impact on both the plasma membrane and the cell wall of *S. cerevisiae*.

During the course of evolution yeasts adapted to employ the high osmolarity glycerol (HOG) pathway to recover after dangerous cell shape modifications and intracellular water disbalance caused by environmental osmotic pressure changes^17,18^. The HOG pathway controls cell volume restoration by employing several mechanisms: changes in enzyme activities, cell cycle arrest, glycerol channel closure and gene expression^19^ as well as metabolic adaptation processes leading to both production and retention of the osmolyte glycerol yield^17,19^. The best-studied regulator of this process is the mitogen-activated protein kinase (MAPK) Hog1, which acts as the effector kinase in a highly conserved MAPK cascade^20^. Upon cell shrinkage, Hog1 is phosphorylated and activates transcription, which in turn regulates the production of enzymes producing glycerol as an intracellular osmolyte. In addition, the phosphorylated Hog1 closes the glycerol channel Fps1^18^. Due to glycerol retention the increase in internal osmolarity forces water molecules back into the cell resulting in pre-stress volume restoration. Consequently, deletion of Hog1 results in osmosensitivity^20^.

PEF exposure causes a similar effect as osmotic stress does, in the sense that intracellular water balance is disturbed. Thus, the HOG pathway could be a possible candidate for biochemical recovery after electroporation. Inactivation of the main HOG pathway regulator, the Hog1 MAPK gene was chosen to provide insight into whether yeasts could recover from electroporation without it. Hyperosmotic pressure causes intracellular water molecules to exit the cell which in turn causes it to shrink^21^. On the contrary, hypoosmotic pressure enlarges the cell by filling it with an excessive amount of extracellular water^22^. In both cases intracellular substance concentration and subsequent cell shape changes are a threat to yeasts’ viability and functionality, here HOG pathway steps in the regulation of canal protein movement and regulation of intracellular glycerol concentration reverts the shape changes caused by osmotic stress^18^. To this day there is no described intracellular pathway that is responsible for cell recovery after electroporation. In this study, we set out to employ osmotic shock treatment to elucidate its effect on (1) viability, (2) permeability and recovery of PEF-treated yeast cells as well as (3) to investigate the involvement of HOG in yeast cell responses to PEF and osmotic shock treatments.

## Materials and methods

### Yeast strains

Y00000 (BY4741; MATα; his3Δ1; leu2Δ0; met15Δ0; ura3Δ0) *S. cerevisiae* strain which in this study will be referred to as wild type (WT) and Y02724 (YLR113w; BY4741: MATα; his3Δ1; leu2Δ0; met15Δ0; ura3Δ0; YLR113w::kanMX4) which in this study will be referred to as *Δhog.* Both strains were aquired from Euroscarf, Germany.

### Cultivation and preparation of yeast cells

Cells were grown overnight in shaker TOU-10–2 (MRC Int, Israel) at 200 rpm speed in liquid YPD media (2% glucose (Roth, Germany), 2% peptone from casein (Roth, Germany) and 1% yeast extract (Roth, Germany)) in 30 °C temperature until an optical density of the solution was OD600 = 0.8 − 1.2 (early exponential growth phase). Cell concentration was 4–9 × 10^7^ cells/mL. Optical density was measured using Halo-RB-10 (Dynamica Scientific Ltd., GB) spectrophotometer. Then cells were centrifuged at 5000 rpm (2000 g) for 4 min and resuspended at room temperature in electroporation buffer EPB (20 mM Tris (AppliChem, Germany), 1 M sorbitol (Fisher Scientific, USA), pH 7.4) at a volume rate of 1:1. Centrifugation and resuspension procedure was repeated 3 times. During the experiments cell suspensions were stored on ice.

### PEF generation system and electroporation procedure

A pulse generator assembled in the Center for Physical Sciences and Technology^23^ was used in the experiments. 500 µL of yeast cell suspension was placed into a cuvette with a 0.2 cm gap between electrodes (Fisher Scientific, US) and exposed to a single square-shaped pulse with pulse length of 150 µs and an electric field strength (E) of up to 10 kV/cm.

### Osmotic shock application procedure

5 s after the pulse, to 500 µL electroporated cell suspension 1500 µL of either (1) distilled water, (2) 20 mM Tris, 0.5 M sorbitol, pH 7.4, as a hypoosmotic solution and 3) 20 mM Tris, 1.5 M sorbitol, pH 7.4, (4) 20 mM Tris, 2 M sorbitol, pH 7.4 as a hyperosmotic solution was added. Final sorbitol concentration in respective samples was (1) 0.25 M (π = 6.11 atm), (2) 0.625 M (π = 15.28 atm), (3) 1.375 M (π = 33.62 atm), (4) 1.75 M (π = 42.79 atm). For further reference final sorbitol sample concentration will be used. For viability and intracellular compound leakage experiments suspension then was incubated on ice for 5 min. Turbidity measurements were performed starting from 10 s and fluorescence assay starting from 5 s after PEF was applied. The osmotic pressure of each solution was calculated by:

1 $$\pi =iMRT$$

where *π* is osmotic pressure, *i* is van’t Hoff’s factor (for this solution *i* = 1), *M* is Molar concentration of the solution (mol/L), *R* is the ideal gas constant (0.08206 L atm mol^−1^ K^−1^) and *T* is the temperature in Kelvin (K).

### Evaluation of cell viability

The cell suspension was diluted by performing serial dilutions until overall dilution was 8000 times and plated on solid YPD media (2% glucose (Roth, Germany), 2% peptone from casein (Roth, Germany) and 1% yeast extract (Roth, Germany), 1.2% agar (Merck, Germany)). Plates were incubated for 48 h at 30 °C temperature in INCU-Line (VWR, USA) incubator. After incubation, the number of colony-forming units (CFU) was counted. A viability of 100% corresponds to the number of CFU formed by untreated cell suspension.

### Evaluation of intracellular component leakage

Pre-treated cells were removed by centrifugation (4 min at 5000 rpm speed (2000 g)). 30 µL of supernatant was added to 1500 µL of Coomassie brilliant blue dye (Biorad, USA) and incubated for 5 min at room temperature. After incubation, solution absorption λ = 595 nm was measured using Halo-RB-10 (Dynamica Scientific Ltd., GB) spectrophotometer. Protein concentration was calculated by using BSA (Sigma Aldrich, Germany) standard (0.5–2 mg/mL) calibration curve. Nucleic acid and tyrosine/tryptophan amino acid containing compound efflux was measured using the same spectrophotometer at 260 nm and 280 nm wavelengths respectively.

### Evaluation of cell size

The yeast cell suspension was transferred to BD PhoenixSpec Nephelometer (BD, Canada) and turbidity of the solution was measured over time steps of 10, 30, 40, 90, 180, and 300 s, then 10 and 30 min.

Cell size was evaluated according to D. H. Melik and H. S. Fogler, where turbidity (*T*) of a monodisperse system of non-absorbing isotropic spheres is given by:

2 $$T =\pi N{r}^{2}Q$$

where *N* is particle concentration, *r* is particle radius and Q is the scattering coefficient^24^. Baker’s yeast *Q* is equal to 0.158 ± 0.005 cm^−1^(mg dry wt/mL)^-1^ according to B. Beauvoit et al.^25^.

### Evaluation of membrane recovery

The osmotic shock was applied to PEF-treated cells as mentioned above. Every minute, up to 9 min after PEF, a 199 µL sample was taken and transferred to fluorescence measurement cuvettes where 1 µL of 0.2 mM Sytox green nucleic acid strain dye (Invitrogen Thermofisher Scientific, USA) solution in DMSO was added. The dye concentration at the time of measurement was 1 µM. Measurements were done using PerkinElmer luminescence spectrometer LS 50B (SpectraLab Scientific inc., Canada). Results were obtained by recording fluorescence intensity value at 525 nm with excitation at 490 nm as per manufacturers’ instructions.

### Statistical analysis

Mean, standard deviation and p-values of ANOVA single factor test from 3 independent experiment repeats were calculated using the Microsoft Excel program Version 2207 (Build 15,427.20210 Click-to-Run).

## Results

### PEF effect on *S. cerevisiae* cells viability

**Figure 1:**
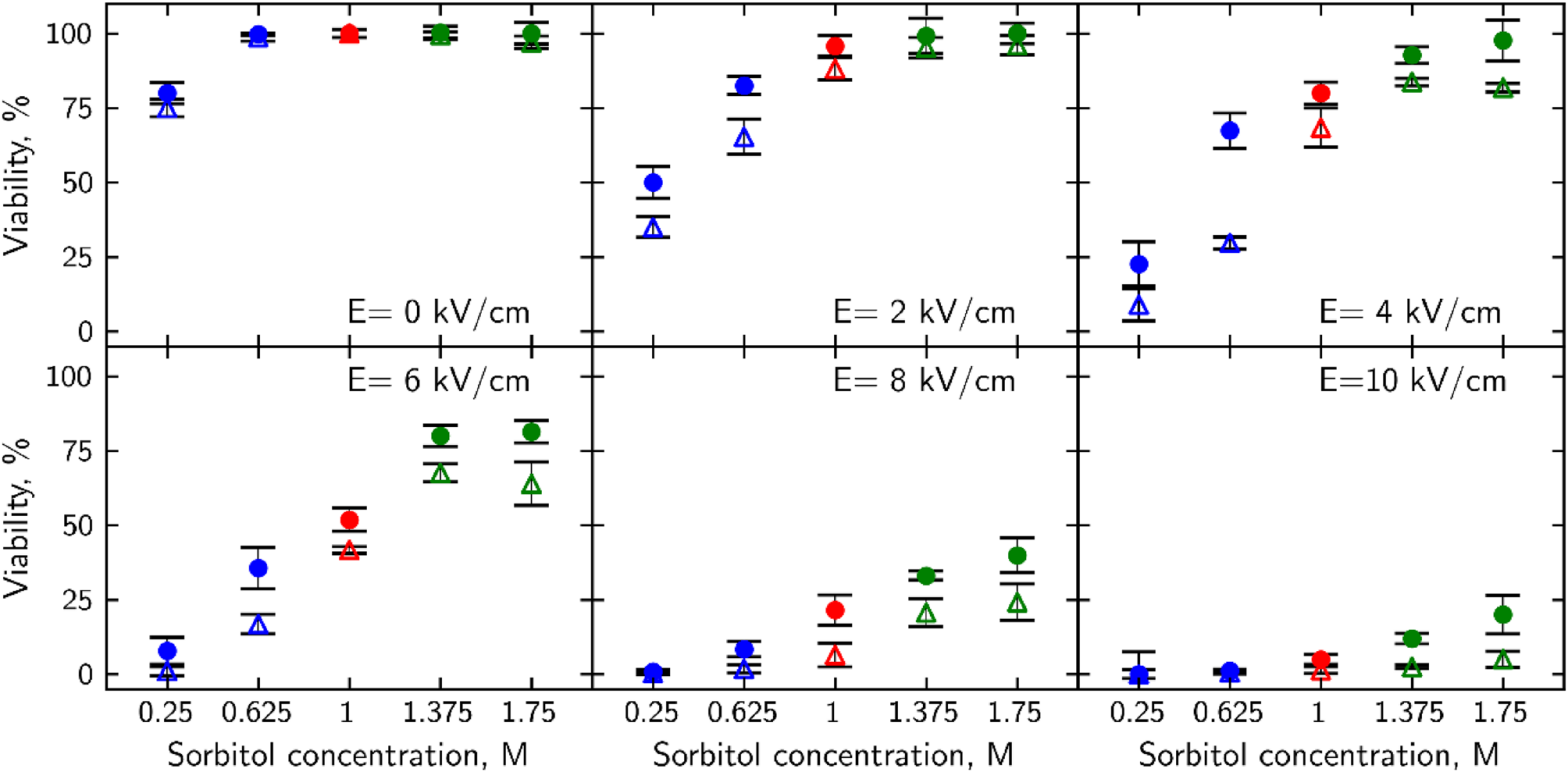
Osmotic shock effects on the viability of PEF-treated yeast cells. WT cells are depicted as filled circles, *Δhog* as hollow triangles. Hypoosmotic shock (0.25 M and 0.625 M sorbitol) is depicted in blue, isoosmotic (1 M sorbitol) in red, and hyperosmotic (1.375 M and 1.75 M sorbitol) in green. p-values can be found in Supplementary Table 1.

To create a baseline yeast response to PEF research was started by analyzing 150 µs single pulse of 2, 4, 6, 8 and 10 kV/cm strength effects on yeast cell viability without additional osmotic shock treatment. Experiments were carried out in a 1 M sorbtol-containing environment which is considered iso-osmotic conditions (red symbols throughout the article). It was shown (Fig. 1) that with increasing field strength, the viability of cells from both strains decreases with a maximum decrease in viability observed after 10 kV/cm PEF treatment: for wild-type yeast 5 ± 1.7% viability and for *Δhog* − 1.4 ± 1.1%. After weak field application of 2 kV/cm wild type yeast cell viability only decreased by 4 ± 3.4%, *Δhog* − 12 ± 3.9%. Furthermore, the viability of *Δhog* cells was 10–20% more impacted by PEF of all strengths compared to WT cells. Such results correlate with other studies^26,27^. Researchers show that with increasing pulsed electric field strength application more damage to the plasma membrane is induced which decreases viability.

### Osmotic shock effect on electroporated *S. cerevisiae* cells viability

To investigate whether PEF-induced decrease in the viability can be altered by subsequent changes in the osmolarity of media, cells were transferred into either hyper- (1.375 M or 1.75 M sorbitol) or hypoosmotic (0.25 M or 0.625 M sorbitol) conditions 5 s after PEF application and incubated for 5 min. A sudden change in the osmolarity of the media was defined as osmotic shock treatment. Viability results (Fig. 1) indicate that a higher count of yeast cells incubated under hyperosmotic conditions after PEF treatment retained their viability in comparison to cells incubated under iso-osmotic (non-changed) conditions. Thus, hyperosmotic shock treatment reduced the severity of the impact of PEF on yeast viability: WT strain results show that it could be fully restored after 2 kV/cm PEF strength with subsequent 1.375 M hyperosmotic shock and after 4 kV/cm PEF strength with 1.75 M shock. A similar but weaker response was observed in *Δhog* cells. Incubation in hyperosmotic conditions after PEF increased viability in all field strength application cases, but complete recovery was achieved only after a 2 kV/cm pulse. After hyperosmotic shock treatment, *Δhog* cells maintained increased viability up to 29% for all PEF strengths applied.

The hypoosmotic shock had the opposite impact on cell viability, it amplified PEF damage. After incubation, the viability of wild-type cells in 0.625 M sorbitol solution after 6 kV/cm electric field strength dropped to 36 ± 6.6%, for *Δhog* − to 17 ± 3.4% when at the same electric field strength without osmotic shock viability was 52 ± 3.9% and 42 ± 1.1% respectively. Further decrease in sorbitol concentration with the same 6 kV/cm field reduced wild-type viability by more than 90% and almost completely eradicated *Δhog* cells. We can see that by applying different osmolarity solutions post-PEF it is possible to increase or reduce yeast cell viability.

Although in both strains’ similar trends of higher viability after hyperosmotic shock treatment and decrease after hypoosmotic shock treatment were observed, HOG pathway inactivation significantly (p > 0.05) reduced the ability of colony unit formation.

### Osmotic shock effect on the intracellular compound leakage

**Figure 2:**
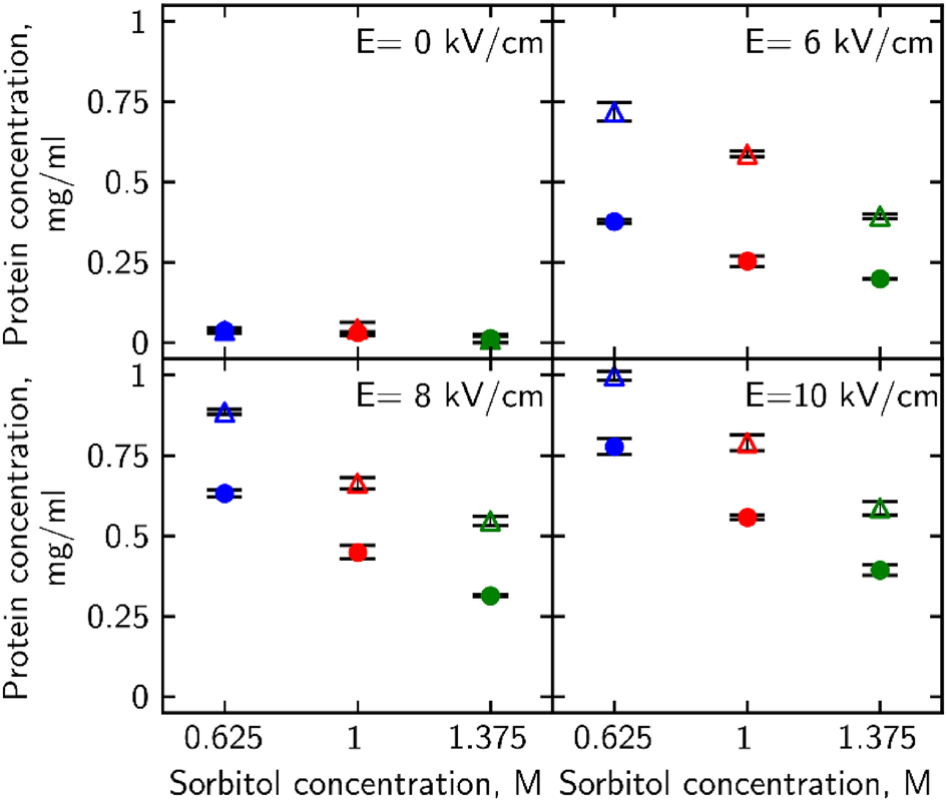
Protein amount in the media of PEF (2–10 kV/cm strength) and osmotic shock-treated yeast cells. Protein concentration values for WT cells are depicted as filled circles, for *Δhog* – as hollow triangles. Hypoosmotic shock (0.625 M sorbitol) is depicted in blue, iso-osmotic (1 M sorbitol) in red and hyperosmotic (1.375 M sorbitol) in green. p-values can be found in Supplementary Table 2.

Permeability changes were assessed to investigate further the full scale of PEF and osmotic shock treatments on yeast cells. Research has indicated that an increase in plasma membrane permeability can be assessed by observing the exchange of molecules either out of (as demonstrated in Aronson et al. study^28^) or into (as demonstrated in Nowosad et al. study^29^) the cell, which is typically not possible under normal resting conditions. To provide insight into yeast cell permeability changes after PEF and subsequent osmotic shock application, several intracellular compound leakages into the media were measured. Intracellular protein concentration in media was measured using the Bradford method^30^. It is important to note that Bradford’s measurement method did not distinguish between intracellular proteins and proteins that got into the media via the distruction of outer layers after electroporation. Despite this, since the protein measurements exhibit a similar pattern to the other measured compounds, it is possible to draw conclusions regarding cell permeability. Results were as follows (Fig. 2). Firstly, the increase in the electric field strength resulted in a higher concentration of protein molecules in the media. After 6, 8, and 10 kV/cm strength pulses for wild-type cells concentration of proteins in the media was 0.25 ± 0.017, 0.45 ± 0.02 and 0.56 ± 0.007 mg/mL; for *Δhog* − 0.59 ± 0.009, 0.66 ± 0.017 and 0.79 ± 0.024 mg/mL. These results show that *Δhog* cells released more intracellular protein molecules into the media than wild-type cells. Secondly, incubation in hyperosmotic (1.375 M sorbitol) conditions after PEF treatment decreased protein concentration in the media. It was reduced by approximately 21, 30 and 29.5% for wild-type cells and *Δhog* ~ 33, 18 and 26%. Thirdly, incubation in hypoosmotic (0.625 M sorbitol) conditions increased protein concentration in the media for wild-type cells by about 49, 41, 39%, and for *Δhog* ~ 22, 33 and 26% compared to isoosmotic conditions.

**Figure 3:**
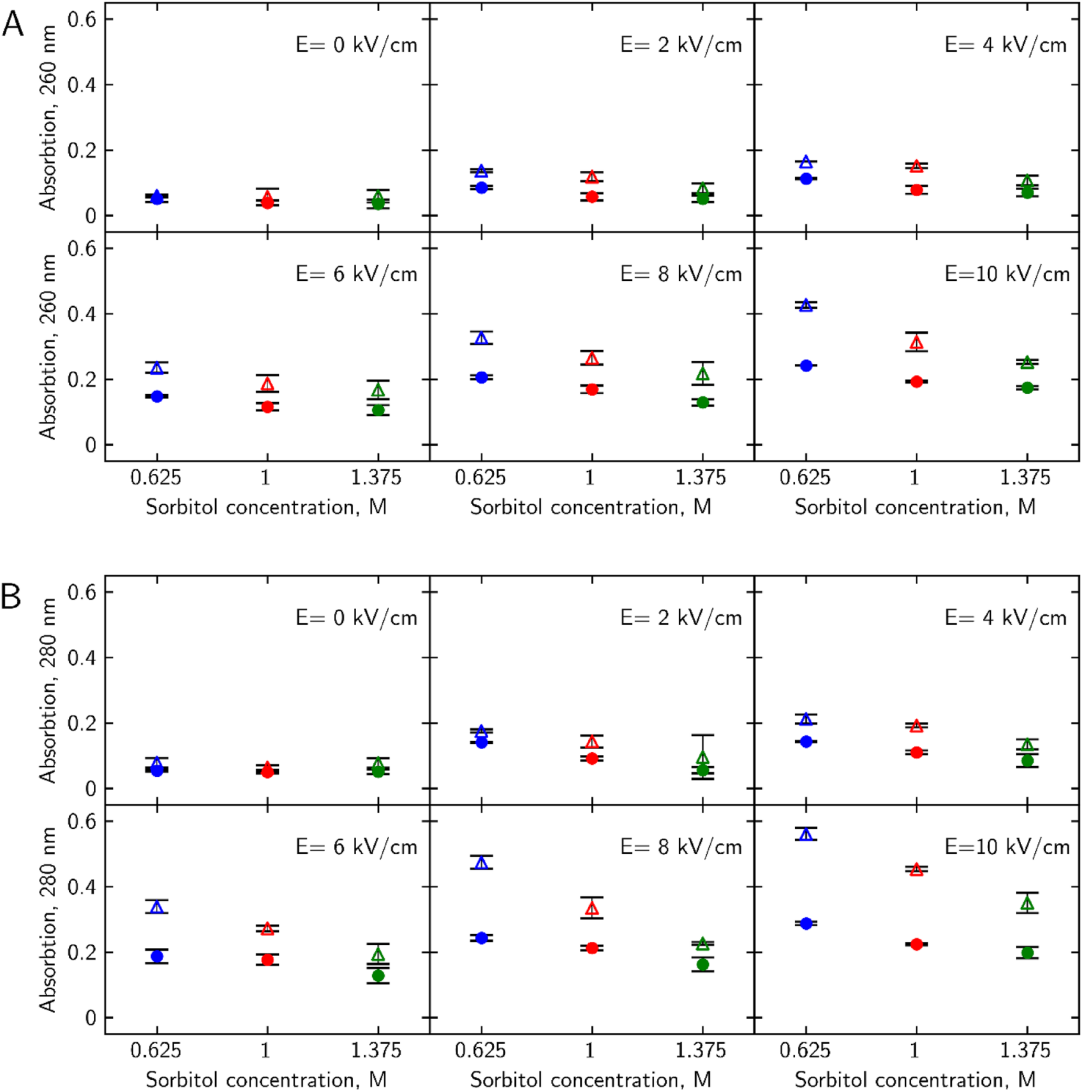
Osmotic shock after PEF effect on A 260 nm and B 280 nm light absorbing compound efflux into the media. WT cells are depicted as filled circles, *Δhog* as hollow triangles. Hypoosmotic shock (0.625 M sorbitol) is depicted in blue, iso-osmotic (1 M sorbitol) in red, and hyperosmotic (1.375 M sorbitol) in green. p-values can be found in Supplementary Table 3.

Compounds that absorb UV light at 260 nm (attributed to nucleic acids^31^) and 280 nm (attributed to tyrosine and tryptophan amino acids^32^) efflux into the media was measured (Fig. 3.). Hypoosmotic shock treatment increased these compound amount by ~ 10% for wild type cells and by 10–25% for *Δhog* cells. Hyperosmotic shock reduced that amount by 5–10% for WT and by 5–20% for *Δhog* cells.

Post-PEF application of hyperosmotic shock treatment has reduced and hypoosmotic shock—increased the efflux of intracellular compounds into the media after PEF treatment for all cases studied. *Δhog* yeasts released about 10–20% more compounds which indicates the lesser ability to retain important molecules for survival within the cell itself. Thus, *Δhog* were more severely impacted by the treatments, indicating HOG pathways’ importance in the recovery, survival, and vital compound retention of yeast cells.

### Osmotic shock effect on yeast cell size

**Figure 4:**
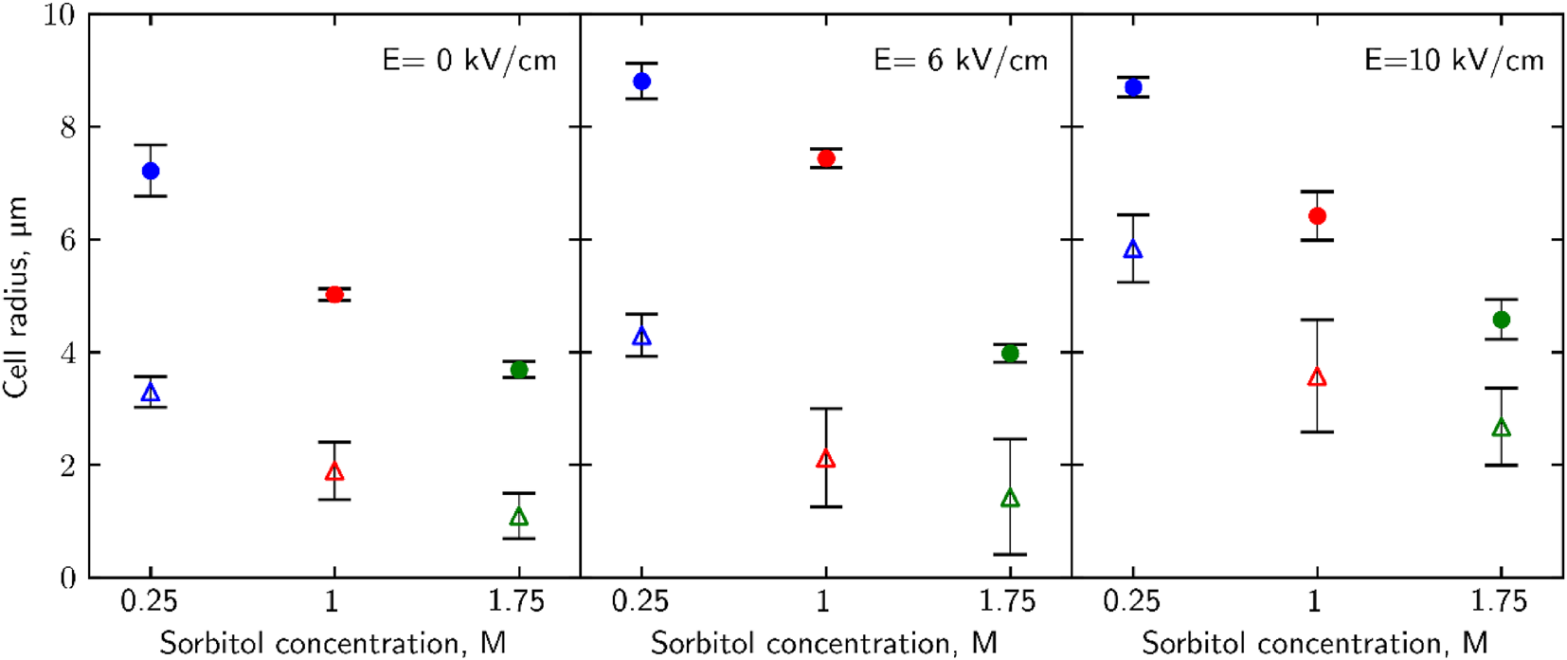
Osmotic shock after 6–10 kV/cm strength PEF effect on wild type and *∆hog* cell radius. WT cells are depicted as filled circles, *Δhog* as hollow triangles. Cell radius values at 10 s after the electric pulse are displayed. Hypoosmotic shock (0.25 M sorbitol) is depicted in blue, iso-osmotic (1 M sorbitol) in red, and hyperosmotic (1.75 M sorbitol) in green. p-values can be found in Supplementary Table 4.

In order to shed light on the mechanisms behind the substantial impact of osmotic shock on cell viability and loss of intracellular compounds following PEF treatment, the change in the radius of yeast cells was assessed after these treatments. At rest conditions yeast cell radius is about 4–5 µm (Fig. 4) which corresponds to information found in literature^33^. Results show that pulsed electric field application increases wild-type cells’ radius by as much as 2.4 µm (from 5.0 ± 0.1 µm to 7.4 ± 0.16 µm), although there were no significant differences between 6 and 10 kV/cm fields strengths. In combination with post-PEF of 6 kV/cm strength and hypoosmotic shock treatment, the cell radius increased to 8.8 ± 0.31 µm. On the contrary, after hyperosmotic shock, the cell radius is reduced back to rest conditions, 3.9 ± 0.16 µm. Also, cell radius changes can be observed without the application of PEF: hypoosmotic shock by itself increases wild-type cell radius to 7.2 ± 0.46 µm; hyperosmotic—decrease to 3.7 ± 0.14 µm. Similar effects are observed in *∆hog* cells, albeit in rest conditions cells are noticeably smaller, having a 1.9 ± 0.51 µm radius. 10 kV/cm strength pulse increases their size to 3.6 ± 1.00 µm. Radius can be further increased to 5.8 ± 0.60 µm via hypoosmotic shock treatment after 10 kV/cm PEF and decreased to 2.7 ± 0.68 µm with hyperosmotic shock treatment.

Observation of turbidity up to 30 min after PEF application does not reveal any insight into whether cells revert their size back to rest conditions (data not shown). While the immediate effects of PEF and osmotic shock treatments on cell size could be measured, cell size recovery was not detected within 30 min after treatments. The absence of restoration of cell size may be due to cells giving priority to the recovery of the membrane over the recovery of volume. Sudden volume changes could have teared up intacellular fibers or microtubules which form yeasts ‘ cytoskeleton^34,35^ and result in lack of volume regulation. Thus, while the immediate effect of treatments on cell size is apparent, revertion to usual volume was not detected.

### Plasma membrane recovery after PEF and osmotic shock treatment

**Figure 5:**
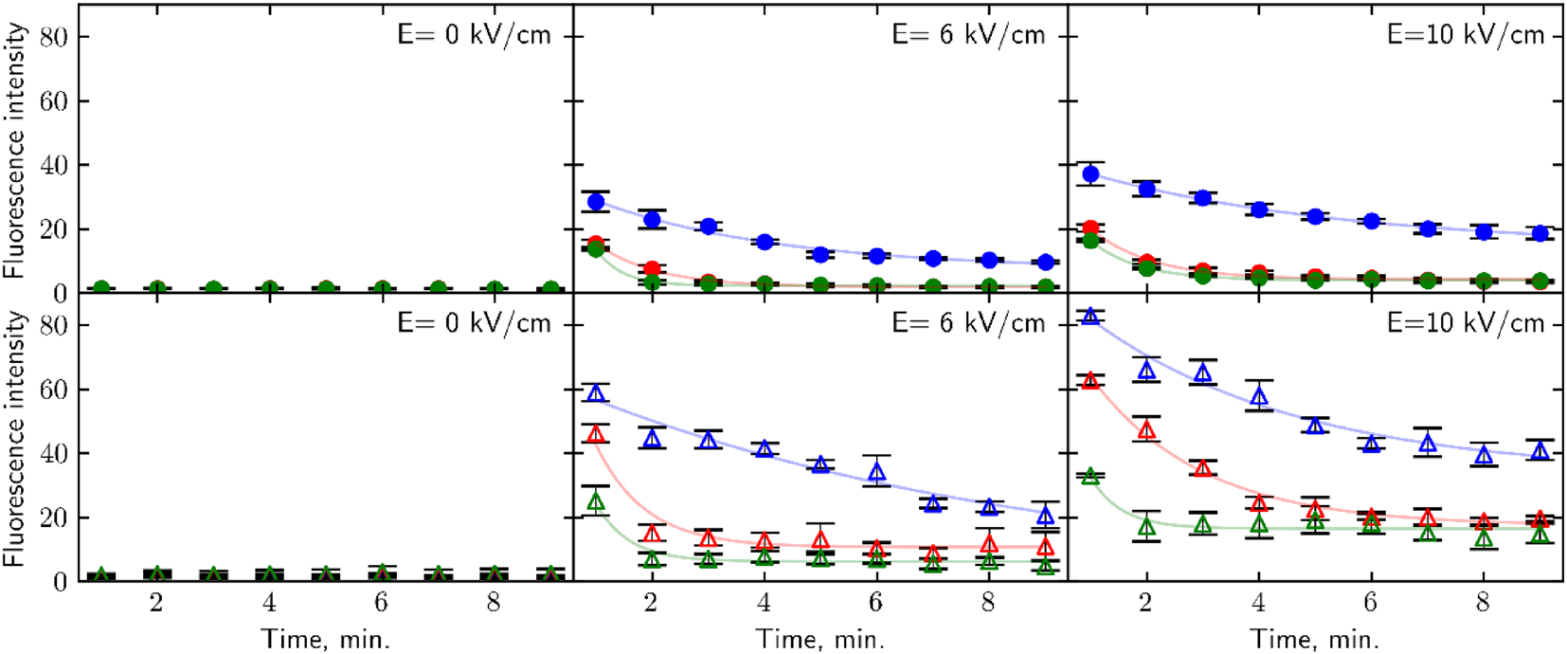
Fluorescence intensity dependence on time after PEF. Hypoosmotic shock (0.25 M sorbitol) is depicted in blue, iso-osmotic (1 M sorbitol) in red, and hyperosmotic (1.75 M sorbitol) in green. WT strain is marked by circles, *∆hog* in triangles. p-values can be found in Supplementary Table 5. R^2^ values of exponential curve fits can be found in Supplementary Table 6.

Plasma membrane recovery was assessed using SYTOX Green dye for fluorescence intensity (FI) measurements. Figure 5 shows the intensity of the fluorescent spectrum peak value (525 nm) dependence on the time of the SYTOX Green addition to the cell suspension after PEF.

The experimental data presented in Fig. 5 shows that FI drops with the increase of time after cell treatment by PEF down to the level of the residual intensity (*I*_*r*_). The total decay time showing cell membrane relaxation to their initial state after PEF action is approximately 120 s and the fluorescence intensity decay can be fitted by the exponential expression^9^:

3 $$I ={I}_{0}*exp\left.\left(- \frac{t}{{\tau }_{l}}\right.\right)+{I}_{r}$$

Here *I*_*0*_ is the fluorescence intensity at *t* = 0 min and *τ*_*l*_ is the characteristic decay time of the lipidic pores. The fitting results show that the permeability of a significant part of the cell membrane recovers to its initial state in a characteristic time *τ*_*l*_ ≈ 1 min for both strains without osmotic shock application. From Fig. 5 we can see that membrane permeability and recovery are greatly affected by the increase in electric field strength and by osmotic shock applied after the pulse.

From these results, FI plateau values after 6 kV/cm strength PEF impulse are within value limits of non-affected yeasts. Thus, the 6 kV/cm electric field strength impulse is causing reversible damage to both yeast strains. In both strain cases cell membrane in iso-osmotic conditions recovers after *τ*_*l*_ ≈ 1 min after PEF is applied. It is clear that *∆hog* cells exhibit greater permeability than wild-type cells, as indicated by their maximum fluorescence intensity (FI) values of 46 and 15 FI units, respectively. The application of hyperosmotic shock resulted in a decrease in both strains the recovery time of the membrane from approximately 1 min to approximately 30 s after PEF and the peak intensity of permeability. Hypoosmotic shock drastically increased both strains’ permeability, WT cells to 28.5 and *∆hog* to 59 FI units and the recovery rate. Wild-type cells’ *τ*_*l*_ ≈ 3.3 min which indicates that characteristic recovery time was increased three times relative to isoosmotic conditions. *∆hog* cells showed *τ*_*l*_ of 8.25 min which is ~ 8 times longer than no osmotic shock treatment. Following treatment with PEF and hypoosmotic shock, the plasma membrane was unable to return to its original state, indicating that the damage to the cells was irreversible. This was evident from the fluorescence intensity (FI) values, which plateaued at approximately 10 FI units for wild-type cells and 24 FI units for *∆hog* cells.

Increase in impulse strength to 10 kV/cm caused irreversible membrane damage to both yeast strains. Without osmotic shock treatment FI stabilizes at 4 units for WT cells and at 19 units for *∆hog* with *τ*_*l*_ ≈ 1 min and *τ*_*l*_ ≈ 2 min, respectively. Hyperosmotic shock decreases recovery time to less than a minute for both strains (*τ*_*l*_ ≈ 50 s for WT and *τ*_*l*_ ≈ 30 s for *∆hog*), however FI remains stable at a higher value (2 FI units for WT, 14 FI units for *∆hog*) than control measurements. Hypoosmotic shock increases membrane permeability and the time it takes for FI to stabilize, *τ*_*l*_ ≈ 4.7 min for wt and *τ*_*l*_ ≈ 3.7 min for *∆hog*. As FI values remain relatively high after stabilization it indicates that cells couldn’t sustain membrane damage.

## Discussion

While electropermeabilization of yeast cells is already applicable in many fields, in optimization of electric field strength, the number of pulses applied and the duration of those pulses usually become the main focus^36^. In our study, permeability results suggest that 6 kV/cm impulse strength causes partially reversible electroporation in yeast cells, and 10 kV/cm impulse causes irreversible damage. According to T. Kotnik et al*.*^37^, there are four ranges of electric field strength, each characterized by properties of the pores formed and subsequent molecular transport. In the range of no detectable electroporation, even if the pores are formed, they are too small and short-lived for measurable molecular transport. In reversible electroporation, a temporary pathway for transport is created, but after the electric pulse, they gradually reseal, the transport ceases, and most cells retain their viability. In the range of non-thermal irreversible electroporation, most pores either do not reseal or reseal too slowly to preserve cell viability. Thus, cells gradually disintegrate and release their intracellular contents into the media. However, these contents are not thermally damaged. Finally, in the range of irreversible electroporation with thermal damage, electric current causes a temperature increase sufficient to cause thermal damage both to the cells to facilitate their disintegration and to the released intracellular molecules. In this study, following T. Kotniks et al*.*^37^ proposed classification, the selected range of 2 to 10 kV/cm electric field strength falls into the categories of reversible electroporation (2–6 kV/cm) and non-thermal irreversible electroporation (6–10 kV/cm). Results confirm that with an increase in electric field strength applied to yeast cells, viability decreases and permeability increases.

**Figure 6:**
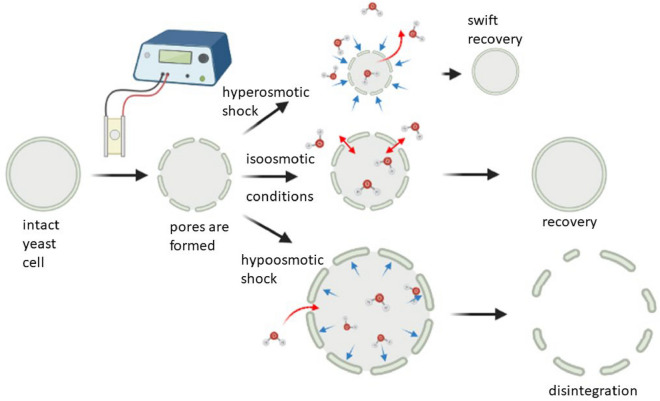
Schematic representation of post-PEF osmotic shock impact on yeast cell. Blue arrows indicate osmotic pressure on the cell exterior. Red arrows show the movement direction of water molecules.

Our study reveals that additional cell treatment in the form of a sudden change in the osmolarity of the media after electroporation makes it possible to change the severity of the damage caused by an electric field with fixed parameters. This knowledge expands the concept of reversible electroporation when reversibility is defined as the lethal outcome of PEF treatment. Our results have shown that not only the electric field strength of 2–10 kV/cm but also the type of sudden media osmolarity change after PEF application determines yeast cell survivability and recovery rate after electroporation (Fig. 6). The rapid increase of sorbitol concentration in the media after PEF treatment increases the survivability of yeast cells. On the contrary, a subsequent decrease of extracellular sorbitol concentration in PEF-treated suspension decreases yeast viability. Sorbitol has already been used in various studies as an osmotic stabiliser^38^. Also, studies have shown that electroporation of Chinese hamster ovary cells depends on buffers' osmolarity^39–41^ where sorbitol influences water movement through the cell membrane. Such water movement, in turn, adds as a component to the plasma membrane recovery or cell disintegration. When electric field application is carried out in hypertonic media, cell permeabilization is observed at a lower voltage than cells maintained in isotonic media and exposed to the same electric pulse parameters. Other factors, such as pH increase after electroporation have shown higher yeast inhibition rates^42^ depicting synergistic effect of PEF with an additional treatment. In addition, a further increase in the osmotic pressure of the post-electroporation media increased the rate of cell resealing^40^. When cell membrane becomes permeabilized, water movement through it becomes easier to achieve. This water movement is the main driving force of the rapid volume change of yeasts. In hypotonic media, water moves into the cell, while in hypertonic media, water moves out of the cell. Since the water cannot be replenished as it is lost or removed as it accumulates, this causes the cells to either mechanically shrink or expand depending on the respective conditions. In another study Guyot et al. link yeast survival and regeneration after osmotic stress to changes in plasma membrane fluidity and subsequent plasma membrane area reduction^43^. Similarly, we propose that plasma membrane area variation due to increased ease of water movement is the reason for different outcomes of post-PEF treatments.

Due to the inability to replace lost water rapidly, cells experience a loss in volume. As a result, the outer layers of the cell also contract, leading to a reduction in both the surface area of the membrane and the size of PEF-induced pores. This contraction makes it easier for the cell to close the pores. Authors note that cell size estimation via turbidity measurement is a crude and not-so-accurate in terms of actual cell size estimation. It still is relevant in showing a trend of change in cell radius after treatments. This calculation method requires to accept an assumption that all cells in the solution are dispersed separately and uniformly which is not what actually happens because yeasts tend to form aggregates. Nakayama et al. showed more accurate microscopic imagery that depicts fusion yeasts swelling after hypoosmotic shock which shows cell size changes in more mild manner^44^. Petelenz-Kurdziel et al*.* state that *S. cerevisiae* cells shrink up to 55% in hyperosmotic shock^45^. Due to information found in literature and theoretical understanding of osmotic stress response, we can state that cell size changes after osmotic shock application does happen. Also, with each increase in electric field strength application theoretically we can assume the corresponding increase in different particles in the solution from the destruction of yeasts. These factors can have an influence on light scattering during turbidity measurements and therefore impact cell radius calculation errors displayed in this study.

As microsecond electric field damage is tied to the outside layers of the cell^46^ we propose that mechanical alteration of cell radius impacts the ability of the cell to regenerate outer layer damage. When cells find themselves in hyperosmotic conditions, they shrink. A decrease in volume happens because of osmotic pressure build-up near the membrane, which forces the solute, in this case, water molecules, to move towards higher soluble molecule concentration outside the plasma membrane^26^ (Fig. 6 red arrows, top row). By transferring electroporated cells into hyperosmotic media after the pulse, forced loss of intracellular water causes the cell to shrink, thus decreasing its plasma membranes' surface area and size of pores. Reduction of the pore size facilitates the membrane's recovery and by extension retainability of cell integrity. Observed decrease in cell size after hyperosmotic shock (Fig. 4) corresponds with viability increase (Fig. 1) and permeability decrease (Figs. 2, 3) after the same treatment for both yeast strains. Similarly, an increase in cell radius (Fig. 4) correlates to viability loss (Fig. 1) and an increase in intracellular compound amount outside of the cell (Figs. 2, 3), supporting the idea. A study of *S. pombe* yeasts showed that post-pulse incubation in 2 M (hyperosmotic) sorbitol solution increased transformation efficiency by as much as an order of magnitude^47^. Such observation agrees with our results showing that more cells survived the treatment due to hyperosmotic stress endured after PEF. Our results demonstrate that post-PEF hyperosmotic shock not only enhanced cell survival but also accelerated the rate of plasma membrane recovery (Fig. 5). It is important to note that not all lethal damage is caused by permeability. During exposure to PEF structure, some molecules can be damaged due to oxidative stress or even directly by an electric field^26^. Changes in osmotic conditions are incapable of compensating for such injuries, and therefore, we consider them irreversible. On the contrary, hypoosmotic stress causes cells to swell^22^. Other studies also showed the increase in cells` size during electroporation^40^. So, in combination with both treatments hypoosmotic shock after electroporation causes increased uptake of extracellular media into the already swollen cell (Fig. 6 bottom row). Excessive mechanical damage from the built-up intracellular water pressure can subsequently cause the membrane to burst^48^. In another study, electroporation has already been shown to amplify high-pressure carbon dioxide (HPCD) pasteurization effectiveness when combining both treatments^49^. Thus, hypoosmotic shock application could further assist in optimizing damaging effects on the cell during pasteurization processes.

It is evident that the measurable and observable effect of osmotic media composition has occurred both during and after electric pulse application. When an electric field is applied, and the plasma membrane becomes more permeable, extracellular water molecules via diffusion are transferred from the extracellular solution to the inside of the cell. Such water movement changes are attributed to general hypoosmotic stress as well. For *S. cerevisiae* cells, this is where the HOG biochemical pathway should be activated^18^. Inactivation of Hog1 leads to osmosensitivity, thus the lack of regenerative properties results in a longer permeable state after electroporation^20^. Because of the longer permeable state, more intracellular molecules could diffuse out of the yeast cell, making it harder for it to retain survivability and fix its membrane. *∆hog* cells displayed significant sensitivity to osmotic shock treatments thus displaying phenotypes consistent with that of lacking HOG pathway activity. Also, *Δhog* mutant yeast cells were measurably smaller before and after treatments (Fig. 4). Results of this study have shown a systemic lack of survivability and regenerative properties displayed by *∆hog* yeasts compared to wild-type cells. In comparison to wild-type cells, more intracellular compounds were found in cell media as well indicating their lack of containment. Because *∆hog* cells displayed significantly worse responses to treatments than wild-type cells, we conclude that HOG pathway is vital in recovery after electroporation. In the future works it is of great importance to name and distinguish exact genetic sequences and biochemical participants of HOG pathway to fully describe its involvement in electroporation recovery process.

Studies of various electroporation treatments can help improve electric field application range, their efficiency and reduce cost. With post-PEF osmotic shock application, it is possible to retain viable cells that were suffering permanent damage otherwise. Such increased viability retainment makes it possible to use higher electric field strength for example to achieve a higher transformation rate without the cell viability loss that comes with it. Hypoosmotic shock application after electroporation could increase the efficiency of electric food pasteurization while decreasing the cost of the whole process by reducing the electric field strength needed to inhibit microorganisms. To summarise, we showed that subsequent change in osmolarity after pulsed electric field treatment could amplify or diminish a pulse's destructive effects.

## Conclusions

Via viability and various plasma membrane permeability experiments, we have shown that Hog1 deficient *S. cerevisiae* cells were considerably more sensitive to electric field treatment, thus linking the involvement of the HOG pathway to recovery after electroporation. By changing the osmolarity of the media after PEF treatment, we influenced the plasma membrane recovery rate, the severity of permeabilization, and the survivability of yeast cells. When the plasma membrane of the cell is electroporated, it becomes more susceptible to water diffusion and subsequent changes in cell shape. This susceptibility can be manipulated by consecutive changes in extracellular osmotic conditions. Which can alter the damage to the plasma membrane caused by PEF. Thus, enhancing both destructive and regenerative electroporation effects. We showed that post-PEF hyperosmotic shock treatment can be used to reverse the damages caused by electroporation, improve *S. cerevisiae* cells viability and fasten the recovery rate of the plasma membrane. On the contrary, hypoosmotic shock improved the lethal effects of PEF treatment resulting in permeabilization and a decrease in the viability after exposure to weaker electric field strengths. As such, we showed that it is possible to disassociate the concept of electroporation reversibility from solely electric field parameters. We conclude that a combination of PEF and post-PEF treatment could lead to much more successful procedure optimization and cost-effective applications.

## Supplementary Information

Supplementary Tables.

## Data Availability

The datasets generated during and/or analysed during the current study are available from the corresponding author on reasonable request.
