# Supplementary material for: Investigation of osmotic shock effect on pulsed electric field treated S. cerevisiae yeast cells
Source: Sci Rep. 2023 Jun 29;13:10573. doi: 10.1038/s41598-023-37719-4 (PMC10310692; doi:10.1038/s41598-023-37719-4)
Supplement: Supplementary file 1 — Supplementary Tables. [file 41598_2023_37719_MOESM1_ESM.docx]

SUPPLEMENTARY INFORMATION

Table 1. p-values of ANOVA single factor test of yeast cell viability values between osmotic shock groups. p-value < 0.05 represents statistical difference between groups.

| Electric field strength (kV/cm) | p-value (for WT cells) | p-value (for *Δhog* cells) |
| --- | --- | --- |
| 0 | 1.17*10^-29^ | 1.46*10^-29^ |
| 2 | 1.89*10^-29^ | 4.44*10^-28^ |
| 4 | 9.2*10^-06^ | 7.58*10^-09^ |
| 6 | 6.33*10^-09^ | 2.03*10^-12^ |
| 8 | 1.4*10^-06^ | 0.000271 |
| 10 | 2.94*10^-22^ | 3.02*10^-19^ |

Table 2. p-values of ANOVA single factor test of protein concentration values between osmotic shock groups. p-value < 0.05 represents statistical difference between groups. p-values without statistical difference between groups are presented in italic.

| Electric field strength (kV/cm) | p-value (for WT cells) | p-value (for *Δhog* cells) |
| --- | --- | --- |
| 0 | *0.736744* | *0.76914* |
| 6 | 0.008267 | 0.007113 |
| 8 | 0.001659 | 0.001659 |
| 10 | 0.002241 | 0.003401 |

Table 3. p-values of ANOVA single factor test of 260 and 280 nm absorption values between osmotic shock groups. p-value < 0.05 represents statistical difference between groups. p-values without statistical difference between groups are presented in italic.

| Electric field strength (kV/cm) | p-value (for WT_260_ cells) | p-value (for *Δhog*_260_ cells) | p-value (for WT_280_ cells) | p-value (for *Δhog*_280_ cells) |
| --- | --- | --- | --- | --- |
| 0 | *0.095174* | *0.660452* | *0.111849* | *0.45528* |
| 2 | 0.000251 | 0.028074 | 0.001784 | 0.017564 |
| 4 | 0.014833 | 0.031512 | 0.000113 | 0.001492 |
| 6 | 0.011395 | *0.08596* | 0.002859 | 0.001021 |
| 8 | 0.001817 | 0.001696 | 0.002352 | 0.002619 |
| 10 | 0.002049 | 0.001569 | 0.013788 | 0.000512 |

Table 4. p-values of ANOVA single factor test of yeast cell radius values between osmotic shock groups. p-value < 0.05 represents statistical difference between groups.

| Electric field strength (kV/cm) | p-value (for WT cells) | p-value (for *Δhog* cells) |
| --- | --- | --- |
| 0 | 0.000385 | 0.000704 |
| 6 | 0.002526 | 3.15*10^-05^ |
| 10 | 0.049298 | 0.000212 |

Table 5. p-values of ANOVA single factor test of fluorescence intensity values between osmotic shock groups. p-value < 0.05 represents statistical difference between groups. p-values without statistical difference between groups are presented in italic.

| Electric field strength (kV/cm) | Time after impulse, min | p-value (for WT cells) | p-value (for *Δhog* cells) |
| --- | --- | --- | --- |
| 0 | 1 | *0.737095* | *0.778087* |
|  | 2 | *0.797853* | *0.881098* |
|  | 3 | *0.550156* | *0.876446* |
|  | 4 | *0.69407* | *0.913361* |
|  | 5 | *0.658421* | *0.73006* |
|  | 6 | *0.182186* | *0.538888* |
|  | 7 | *0.494125* | *0.618089* |
|  | 8 | *0.426818* | *0.652613* |
|  | 9 | *0.578704* | *0.491966* |
| 6 | 1 | 2.45*10^-05^ | 0.000203 |
|  | 2 | 5.97*10^-05^ | 1.57*10^-05^ |
|  | 3 | 6.34*10^-07^ | 7.09*10^-06^ |
|  | 4 | 2.27*10^-07^ | 4.76*10^-06^ |
|  | 5 | 8.13*10^-06^ | 0.000191 |
|  | 6 | 7.65*10^-07^ | 0.000212 |
|  | 7 | 1.34*10^-07^ | 4.44*10^-05^ |
|  | 8 | 3.74*10^-07^ | 0.003536 |
|  | 9 | 4.09*10^-07^ | 0.011644 |
| 10 | 1 | 1.58*10^-05^ | 5.83*10^-08^ |
|  | 2 | 2.3*10^-06^ | 6.87*10^-05^ |
|  | 3 | 7.97*10^-07^ | 1.82*10^-05^ |
|  | 4 | 1.33*10^-06^ | 0.000123 |
|  | 5 | 8.88*10^-08^ | 0.000247 |
|  | 6 | 1.23*10^-07^ | 4.6*10^-05^ |
|  | 7 | 3.38*10^-06^ | 0.000285 |
|  | 8 | 1.98*10^-05^ | 0.000327 |
|  | 9 | 1.48*10^-05^ | 0.000107 |

Table 6. R^2^ values of exponential curve fits shown in fig. 5.

| Curve name | R^2^ value |
| --- | --- |
| WT hipoosmotic 6 kV/cm | 0.980956 |
| WT hipoosmotic 10 kV/cm | 0.980956 |
| WT isoosmotic 6 kV/cm | 0.997158 |
| WT isoosmotic 10 kV/cm | 0.997158 |
| WT hyperosmotic 6 kV/cm | 0.992833 |
| WT hyperosmotic 6 kV/cm | 0.992833 |
| *Δhog* hipoosmotic 6 kV/cm | 0.947515 |
| *Δhog* hipoosmotic 10 kV/cm | 0.967565 |
| *Δhog* isoosmotic 6 kV/cm | 0.945527 |
| *Δhog* isoosmotic 10 kV/cm | 0.989733 |
| *Δhog* hyperosmotic 6 kV/cm | 0.956326 |
| *Δhog* hyperosmotic 6 kV/cm | 0.886954 |
